# Ascites of Unexplained Origin: A Case Report

**DOI:** 10.7759/cureus.23256

**Published:** 2022-03-17

**Authors:** Muteb A Alotaibi, Ahmad M Al Othman

**Affiliations:** 1 Critical Care Department, Prince Mohammed bin Abdulaziz Hospital (PMAH) Second‬⁩ ⁦‪Health‬⁩ ⁦‪Cluster‬⁩ in the Central Region, Riyadh, SAU; 2 Internal Medicine, Prince Mohammed bin Abdulaziz Hospital (PMAH) Second‬⁩ ⁦‪Health‬⁩ ⁦‪Cluster‬⁩ in the Central Region, Riyadh, SAU

**Keywords:** ibd, malignancy, abdominal tb, weight loss, ascites of unexplained origin

## Abstract

Ascites is defined as a massive collection of fluid in the peritoneal cavity, and it is most commonly caused by portal hypertension due to liver cirrhosis, acute liver failure, and heart failure. We report a case of ascites in a 39-year-old male who presented to the ED complaining of abdominal distention, sporadic pain on the epigastric area, unintentional weight loss over the past two months, night sweats, and blood-mixed stool. We conducted several imaging investigations and laboratory studies and none of them revealed any significant findings except for the abdominal CT with contrast, which showed large ascites along with submucosal fat deposition in the long segment of the transverse colon, suggestive of chronic inflammation [probably inflammatory bowel disease (IBD)], omental caking, and thickening. Peritoneal malignancy was highly suspected.

A diagnostic/therapeutic paracentesis was performed, through which 8 L of fluid was drained. Cytology was also performed and this showed mostly reactive mesothelial cells, negative acid-fast bacilli (AFB) culture, and negative polymerase chain reaction (PCR). We also discuss the other investigations performed, none of which helped in establishing a diagnosis.

## Introduction

Ascites is commonly caused by cirrhosis, which leads to an increase in intrahepatic resistance of blood flow, which in turn results in vasodilation of the splenic vessels. Other causes include malignancies such as peritoneal carcinomatosis, hepatocellular carcinoma, and mesothelioma, as well as inflammatory causes such as abdominal tuberculosis, Whipple disease, systemic lupus erythematosus, vasculitis, as well as nephrotic syndrome, ovarian hyperstimulation syndrome, and others [1]. The classification of ascites is based on serum ascites albumin gradient (SAAG) levels: a level of >1.1 g/dL (transudate) indicates portal hypertension with >90% reliability, while a SAAG level of <1.1 g/dL indicates with ~95% accuracy that portal hypertension is not involved [2].

## Case presentation

**Figure 1:**
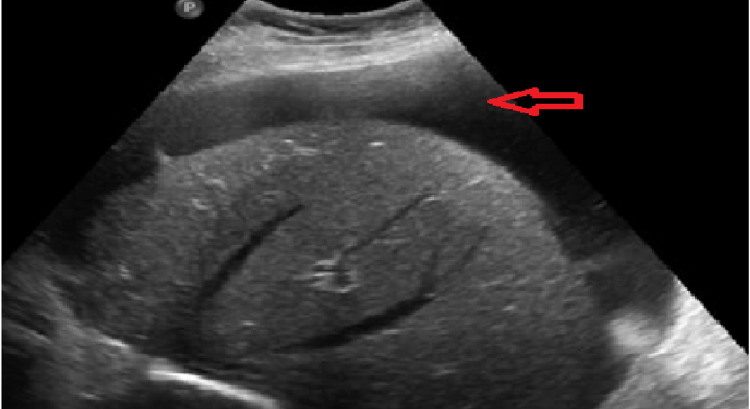
Abdominal ultrasound demonstrating ascitic fluid around the liver (arrow)

A 39-year-old male presented to the ED at our facility with complaints of abdominal distension associated with sporadic epigastric pain, night sweats, unintentional weight loss of about 18 KG over two months, and bright red blood in the stool. The patient's past medical history and family history were unremarkable. He had a surgical history of an application of the stent in the left ureter due to a stone 20 days prior to the admission, and he was on esomeprazole 40 mg OD. The physical examination was uneventful except for the abdominal distension. An ultrasound examination was done at the ED, which revealed moderate abdominal ascites (Figure 1).

**Figure 2:**
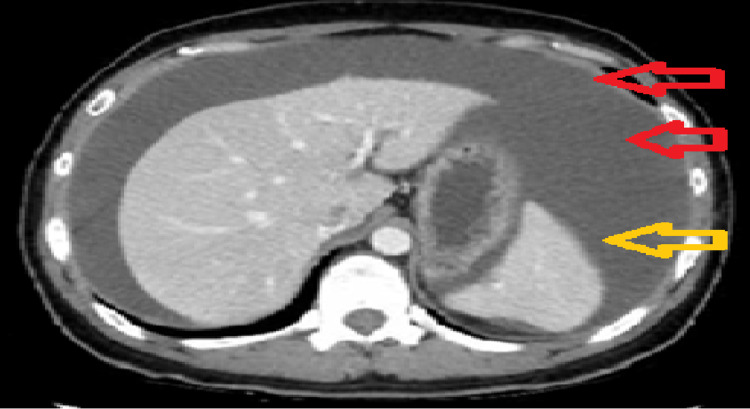
CT abdomen reveals huge ascitic fluid concentration (red arrows) that led to the push-down of the spleen to the level of the backbone (yellow arrow) CT: computed tomography

CT abdomen with contrast showed huge abdominal ascites and submucosal fat deposition of the long segment of the transverse colon, suggestive of chronic inflammation (Figure 2) [3].

Therapeutic and diagnostic peritoneal paracentesis (ascitic tapping) were performed, and about 8 L of fluid was drained, which was clear in color; and smears demonstrated mostly reactive mesothelial cells. The acid-fast bacilli (AFB) culture was negative (no growth), and the same was the case with AFB polymerase chain reaction (PCR) and the culture of the fluid.

Endoscopy and colonoscopy were performed. Upper gastrointestinal (GI) endoscopy revealed a circumferential thickening with an ulcerated surface over the gastric body; multiple biopsies were taken, and histopathological examination indicated mild chronic gastritis. Colonoscopy was introduced to the terminal ileum, and a large pedunculated polyp of about 2 cm in size was removed. The histopathological report showed a tubular adenoma with low-grade dysplasia, but there were no other abnormalities.

Diagnostic laparoscopy was done, and the abdomen was inspected; the whole bowel (mainly large bowel) looked affected and reddish with a fragile surface. The omentum was folded and caking on the transverse colon, and there were no peritoneal deposits. Multiple peritoneal and omental biopsy samples were taken, and the histopathological examination manifested dense fibrous and lobular fatty tissue with scant atypical cells.

## Discussion

Ascites is a common presentation of cirrhosis that develops as a result of long-standing chronic liver injury, which in turn stimulates deformation of the architecture of the liver and fibrosis [1]. A diagnostic abdominal US and CT scans did not show any sign of liver cirrhosis [4]. Also, this was excluded by analyzing the ascitic fluid, which found a high protein concentration of >2.5 g/dL, indicating that the origin of the ascitic fluid was exudate [5]. However, patients’ labs for alanine aminotransferase (ALT) and aspartate aminotransferase (AST) were within normal ranges [1]. Our patient's symptoms included night sweats, considerable unintentional weight loss, and sporadic epigastric pain, in addition to the findings of abdominal CT scan (omental cake) [6,7]. We considered the following differential diagnoses: firstly, one of malignancy (mesothelioma of the peritoneum). Malignant mesotheliomas are tumors that originate from the mesothelial cells and spread throughout the body. The principal sources of origin are the surface linings of three serosal cavities (pleura, peritoneum, and pericardium); it was ruled out by a histopathological study of the peritoneal biopsy [8].

Another possible diagnosis was abdominal and GI tuberculosis (GI TB), which is considered to be an extrapulmonary infection caused by *Mycobacterium tuberculosis* complex, which is responsible for 1-3% of all TB cases in the world and one of the top 10 diseases that leads to death globally. GI TB can develop as a side effect of active pulmonary disease or as a primary infection with no pulmonary involvement. It can affect any part of the GI tract; however, peritoneum and intestine are the most common sites of GI TB involvement [3]; that was excluded based on the negative AFB culture of the peritoneal fluid sample, negative PCR, and AFB tissue culture (which is the gold standard) [9].

We wanted to extend the workup by performing a positron emission tomography (PET) scan, as it is an efficient method for identifying primary cancer or metastasis [10]; unfortunately, that was not available in our health facility. Abdominal CT scan suggested an inflammatory bowel disease (IBD), such as Crohn’s disease and ulcerative colitis. These diseases have similar symptoms. The etiology of IBD is not well known. Generally, the symptoms of IBD include diarrhea, bloody stool, anemia, and arthritis [11]. In order to diagnose IBD, endoscopic and colonoscopy procedures were conducted, and the results demonstrated insignificant findings [11,12].

Upon referring to PubMed and Google Scholar for case reports of similar conditions, we found a single case that is similar to ours; it was reported at the University of Warsaw in Poland. A 31-year-old female with a kidney transplant had ascites of unknown origin; she underwent repetitive paracenteses to drain the fluid, but the ascites eventually resolved on its own [13].

## Conclusions

Ascites is a well-known manifestation of cirrhosis, which was ruled out in our patient by performing ascitic paracentesis; we then opted for a reliable initial diagnostic method along with the imaging technique. Abdominal CT scan that indicated omental cake in addition to the patient's symptoms was suggestive of malignancy or even abdominal TB. Neither of the two could be confirmed by diagnostic techniques. Hence, despite performing all the workups and investigations mentioned above, we could not establish a diagnosis regarding the origin of the ascites. This was, in other words, a case that deceived us.
